# Bis(2,2′-bipyridyl-κ^2^ *N*,*N*′)(sulfato-κ^2^ *O*,*O*′)cobalt(II) ethane-1,2-diol monosolvate

**DOI:** 10.1107/S1600536810050592

**Published:** 2010-12-08

**Authors:** Kai-Long Zhong, Xian-Xiao Pan, Guo-Qing Cao, Lin Chen

**Affiliations:** a Department of Applied Chemistry, Nanjing College of Chemical Technology, Nanjing 210048, People’s Republic of China

## Abstract

The title compound, [Co(SO_4_)(C_10_H_8_N_2_)_2_]·C_2_H_6_O_2_, has the Co^2+^ ion in a distorted octa­hedral CoN_4_O_2_ coordination geometry. A twofold rotation axis passes through the Co and S atoms, and through the mid-point of the C—C bond of the ethane­diol mol­ecule. In the crystal, the [CoSO_4_(C_10_H_8_N_2_)_2_] and C_2_H_6_O_2_ units are held together by a pair of O—H⋯O hydrogen bonds.

## Related literature

For applications of cobalt complexes, see: Bottcher *et al.* (1995 ▶). For related Co compounds with sulfate ions, see: Henning *et al.* (1975 ▶); Lu *et al.* (2006 ▶); Zheng & Lin (2003 ▶); Paul *et al.* (2002 ▶). For isotypic structures, see: Zhong *et al.* (2006 ▶). Zhong (2010*a* ▶,*b* ▶).
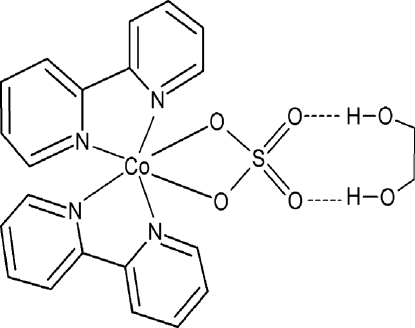


## Experimental

### 

#### Crystal data

[Co(SO_4_)(C_10_H_8_N_2_)_2_]·C_2_H_6_O_2_
*M* *_r_* = 529.44
Monoclinic, 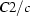

*a* = 16.916 (3) Å
*b* = 11.913 (2) Å
*c* = 12.870 (3) Å
β = 122.16 (3)°
*V* = 2195.6 (10) Å^3^
*Z* = 4
Mo *K*α radiation
μ = 0.93 mm^−1^
*T* = 223 K
0.25 × 0.20 × 0.15 mm

#### Data collection

Rigaku Mercury CCD diffractometer
Absorption correction: multi-scan (*REQAB*; Jacobson, 1998 ▶) *T*_min_ = 0.802, *T*_max_ = 0.874
6197 measured reflections
2509 independent reflections
2153 reflections with *I* > 2σ(*I*)
*R*_int_ = 0.027

#### Refinement

*R*[*F* ^2^ > 2σ(*F* ^2^)] = 0.037
*wR*(*F* ^2^) = 0.085
*S* = 1.06
2509 reflections
155 parameters
H-atom parameters constrained
Δρ_max_ = 0.38 e Å^−3^
Δρ_min_ = −0.36 e Å^−3^

### 

Data collection: *CrystalClear* (Rigaku, 2007 ▶); cell refinement: *CrystalClear*; data reduction: *CrystalClear*; program(s) used to solve structure: *SHELXS97* (Sheldrick, 2008 ▶); program(s) used to refine structure: *SHELXL97* (Sheldrick, 2008 ▶); molecular graphics: *XP* in *SHELXTL* (Sheldrick, 2008 ▶); software used to prepare material for publication: *SHELXTL*.

## Supplementary Material

Crystal structure: contains datablocks global, I. DOI: 10.1107/S1600536810050592/bt5423sup1.cif

Structure factors: contains datablocks I. DOI: 10.1107/S1600536810050592/bt5423Isup2.hkl

Additional supplementary materials:  crystallographic information; 3D view; checkCIF report

## Figures and Tables

**Table:** 

Co1—N1	2.1175 (18)
Co1—N2	2.1285 (17)
Co1—O1	2.1420 (15)
S1—O2	1.4629 (15)
S1—O1	1.4958 (15)

**Table:** 

N1—Co1—N2	76.92 (7)
O1—Co1—O1^i^	66.68 (8)
O2^i^—S1—O2	111.03 (13)
O2—S1—O1	110.97 (9)
O1^i^—S1—O1	103.82 (12)

Symmetry code: (i) 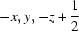.

**Table 2:** Hydrogen-bond geometry (Å, °)

*D*—H⋯*A*	*D*—H	H⋯*A*	*D*⋯*A*	*D*—H⋯*A*
O3—H3⋯O2	0.82	1.97	2.758 (2)	160

**Fig. 1.**
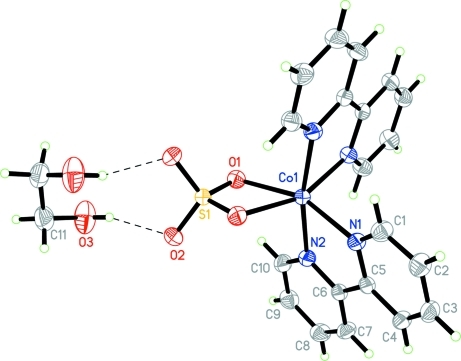
The molecular structure of the title compound showing the atom-numbering scheme and with displacement ellipsoids drawn at the 50% probability level. The light broken lines depict O—H···O interactions. Unlabeled atoms are related to the labelled atoms by the symmetry operator(-x, y, -z + 1/2).

## supplementary crystallographic information

### Comment

Since the first octahedral coordination cobalt complexes was recognized by Werner, some metal cobalt complexes as potent antiviral agents (Bottcher *et al.*, 1995) have been previously reported. Furthermore, many cobalt complexes with monodentate sulfate ions (Henning *et al.*, 1975; Lu *et al.*, 2006), bidentate sulfate ions (Zheng & Lin, 2003) and bidentate bridging sulfate ions (Paul *et al.*, 2002) have been synthesized and characterized. In our investigation, we have carried out solvothermal reactions using metal sulfate and mixed-ligands with the aim  of obtaining complexes retainig some of the solvent molecules capable of hydrogen bonding.

We have previously synthesized Co-complexes with bidentate-chelating sulfate ions, in which uncoordinated O atoms of the sulfate ligand and dihydric alcohol solvent molecules formed classical O—H···O hydrogen bonds *via* a solvothermal reaction, *e.g.* [CoSO_4_(phen)_2_].C_3_H_8_O_2_ (Zhong, 2010*a*), [CoSO_4_(phen)_2_].C_2_H_6_O_2_,  (Zhong *et al.*, 2006).

The title compound crystal structures consist of a neutral monomeric [CoSO_4_(C_10_H_8_N_2_)_2_] complex and a solvent ethane-1,2-diol molecule. The cobalt metal ion is six-coordinated by four N atoms from two 2,2'-bipy ligands and two O atoms from an O,*O*'-bidentate sulfate ion, in a distorted CoN_4_O_2_ octalhedral environment (Fig. 1). The two fairly perpendicularly 2,2'-bipy ligands [dihedral angle = 80.923 (25)°] are in *cis* positions similar to the analogous and [ZnSO_4_(C_10_H_8_N_2_)_2_]. C_2_H_6_O_2_ (Zhong, 2010*b*). The Co—N bond distances, the Co—O bond distances, the N—Co—N bite angle, the O—Co—O bite angle and the dihedral angle between the two chelating NCCN groups is 2.1175 (18)–2.1285 (17) Å, 2.1420 (15) Å, 76.92 (7)°, 66.68 (8)° and 82.798 (73)°, respectively. The [CoSO_4_(C_10_H_8_N_2_)_2_] and C_2_H_6_O_2_ units are connected by a pair of symmetry-related intermolecular O—H···O hydrogen bonds with the uncoordinated O atoms of the sulfate ligand. The Co^2+^ ion, the S atom and the mid-point of C—C bond of the ethane-1,2-diol solvent molecule are located on symmmetry 2 (symmetry code: -*x*, *y*, - *z* + 1/2) (Fig.1 and Table 2).

### Experimental

Orange block-shaped single crystals of the title compound were obtained by a procedure similar to that described previously by Zhong (2010*b*), using CoSO_4_.7H_2_O instead of ZnSO_4_.7H_2_O.

### Refinement

All H atoms were positioned geometrically and allowed to ride on their attached atoms, with C—H =0.93-0.97 Å O—H =0.82 Å and *U*_iso_(H) = 1.2*U*_eq_(C) and 1.5*U*_eq_(O).

### Crystal data

**Table:**

[Co(SO_4_)(C_10_H_8_N_2_)_2_]·C_2_H_6_O_2_	*F*(000) = 1092
*M**_r_* = 529.44	*D*_x_ = 1.602 Mg m^−^^3^
Monoclinic, *C*2/*c*	Mo *K*α radiation, λ = 0.71073 Å
Hall symbol: -C 2yc	Cell parameters from 4711 reflections
*a* = 16.916 (3) Å	θ = 3.3–27.5°
*b* = 11.913 (2) Å	µ = 0.93 mm^−^^1^
*c* = 12.870 (3) Å	*T* = 223 K
β = 122.16 (3)°	Block, orange
*V* = 2195.6 (10) Å^3^	0.25 × 0.20 × 0.15 mm
*Z* = 4	

### Data collection

**Table:**

Rigaku Mercury CCD diffractometer	2509 independent reflections
Radiation source: fine-focus sealed tube	2153 reflections with *I* > 2σ(*I*)
Graphite Monochromator	*R*_int_ = 0.027
Detector resolution: 28.5714 pixels mm^-1^	θ_max_ = 27.5°, θ_min_ = 3.4°
ω scans	*h* = −21→18
Absorption correction: multi-scan (REQAB; Jacobson, 1998)	*k* = −15→12
*T*_min_ = 0.802, *T*_max_ = 0.874	*l* = −12→16
6197 measured reflections	

### Refinement

**Table:**

Refinement on *F*^2^	Primary atom site location: structure-invariant direct methods
Least-squares matrix: full	Secondary atom site location: difference Fourier map
*R*[*F*^2^ > 2σ(*F*^2^)] = 0.037	Hydrogen site location: inferred from neighbouring sites
*wR*(*F*^2^) = 0.085	H-atom parameters constrained
*S* = 1.06	*w* = 1/[σ^2^(*F*_o_^2^) + (0.042*P*)^2^ + 1.1555*P*] where *P* = (*F*_o_^2^ + 2*F*_c_^2^)/3
2509 reflections	(Δ/σ)_max_ < 0.001
155 parameters	Δρ_max_ = 0.38 e Å^−^^3^
0 restraints	Δρ_min_ = −0.36 e Å^−^^3^

### Special details

**Table:**

Geometry. All e.s.d.'s (except the e.s.d. in the dihedral angle between two l.s. planes) are estimated using the full covariance matrix. The cell e.s.d.'s are taken into account individually in the estimation of e.s.d.'s in distances, angles and torsion angles; correlations between e.s.d.'s in cell parameters are only used when they are defined by crystal symmetry. An approximate (isotropic) treatment of cell e.s.d.'s is used for estimating e.s.d.'s involving l.s. planes.
Refinement. Refinement of *F*^2^ against ALL reflections. The weighted *R*-factor *wR* and goodness of fit *S* are based on *F*^2^, conventional *R*-factors *R* are based on *F*, with *F* set to zero for negative *F*^2^. The threshold expression of *F*^2^ > σ(*F*^2^) is used only for calculating *R*-factors(gt) *etc*. and is not relevant to the choice of reflections for refinement. *R*-factors based on *F*^2^ are statistically about twice as large as those based on *F*, and *R*- factors based on ALL data will be even larger.

### Fractional atomic coordinates and isotropic or equivalent isotropic displacement parameters (Å^2^)

**Table:**

	*x*	*y*	*z*	*U*_iso_*/*U*_eq_	
Co1	0.0000	0.19027 (3)	0.2500	0.02208 (12)	
S1	0.0000	−0.03740 (6)	0.2500	0.02590 (17)	
O2	0.06779 (10)	−0.10692 (12)	0.24186 (15)	0.0364 (4)	
O1	−0.04964 (9)	0.04006 (12)	0.14236 (13)	0.0294 (3)	
N2	0.10048 (11)	0.20811 (14)	0.19955 (16)	0.0252 (4)	
N1	0.09694 (11)	0.30564 (13)	0.38131 (15)	0.0245 (4)	
C10	0.09756 (15)	0.15529 (18)	0.1057 (2)	0.0301 (5)	
H10A	0.0499	0.1039	0.0605	0.036*	
C8	0.23390 (15)	0.2495 (2)	0.1420 (2)	0.0348 (5)	
H8A	0.2787	0.2634	0.1226	0.042*	
C1	0.09295 (15)	0.34961 (19)	0.4742 (2)	0.0313 (5)	
H1A	0.0431	0.3300	0.4820	0.038*	
C6	0.17071 (13)	0.28155 (16)	0.26698 (19)	0.0246 (4)	
C3	0.23441 (15)	0.45087 (18)	0.5493 (2)	0.0321 (5)	
H3A	0.2806	0.4992	0.6057	0.038*	
C9	0.16241 (16)	0.17394 (19)	0.0733 (2)	0.0334 (5)	
H9A	0.1581	0.1367	0.0069	0.040*	
C4	0.23959 (14)	0.40584 (17)	0.45416 (19)	0.0284 (4)	
H4A	0.2896	0.4236	0.4460	0.034*	
C5	0.16990 (13)	0.33406 (16)	0.37080 (18)	0.0231 (4)	
C7	0.23820 (15)	0.30389 (18)	0.2396 (2)	0.0301 (5)	
H7A	0.2858	0.3550	0.2864	0.036*	
C2	0.15949 (16)	0.42283 (19)	0.5591 (2)	0.0335 (5)	
H2A	0.1538	0.4526	0.6216	0.040*	
O3	0.01928 (16)	−0.32127 (14)	0.14765 (18)	0.0554 (5)	
H3	0.0241	−0.2604	0.1804	0.083*	
C11	0.03386 (18)	−0.4076 (2)	0.2296 (3)	0.0446 (6)	
H11A	0.0966	−0.4010	0.3013	0.054*	
H11B	0.0297	−0.4790	0.1908	0.054*	

### Atomic displacement parameters (Å^2^)

**Table:**

	*U*^11^	*U*^22^	*U*^33^	*U*^12^	*U*^13^	*U*^23^
Co1	0.0199 (2)	0.0207 (2)	0.0263 (2)	0.000	0.01267 (16)	0.000
S1	0.0213 (3)	0.0204 (3)	0.0355 (4)	0.000	0.0148 (3)	0.000
O2	0.0300 (8)	0.0279 (8)	0.0558 (10)	0.0043 (7)	0.0258 (7)	−0.0023 (7)
O1	0.0258 (7)	0.0271 (7)	0.0308 (8)	0.0010 (6)	0.0120 (6)	0.0008 (6)
N2	0.0231 (8)	0.0252 (9)	0.0274 (9)	−0.0002 (7)	0.0136 (7)	−0.0020 (7)
N1	0.0232 (8)	0.0244 (9)	0.0270 (9)	−0.0012 (7)	0.0140 (7)	0.0004 (7)
C10	0.0299 (10)	0.0289 (10)	0.0334 (11)	−0.0024 (9)	0.0181 (9)	−0.0054 (9)
C8	0.0318 (11)	0.0389 (13)	0.0410 (13)	0.0008 (10)	0.0242 (10)	0.0046 (10)
C1	0.0309 (11)	0.0347 (11)	0.0307 (11)	−0.0035 (10)	0.0181 (9)	−0.0023 (9)
C6	0.0231 (9)	0.0210 (9)	0.0288 (10)	0.0020 (8)	0.0133 (8)	0.0042 (8)
C3	0.0301 (11)	0.0273 (11)	0.0299 (11)	−0.0039 (9)	0.0100 (9)	−0.0040 (9)
C9	0.0371 (11)	0.0351 (12)	0.0361 (12)	0.0033 (10)	0.0249 (10)	−0.0014 (10)
C4	0.0250 (10)	0.0259 (10)	0.0316 (11)	−0.0029 (9)	0.0133 (9)	0.0003 (9)
C5	0.0225 (9)	0.0200 (9)	0.0251 (10)	0.0022 (8)	0.0116 (8)	0.0038 (7)
C7	0.0257 (10)	0.0318 (11)	0.0349 (12)	−0.0051 (9)	0.0175 (9)	0.0008 (9)
C2	0.0371 (11)	0.0348 (12)	0.0279 (11)	−0.0034 (10)	0.0169 (9)	−0.0053 (9)
O3	0.0969 (16)	0.0373 (10)	0.0538 (12)	−0.0015 (10)	0.0548 (12)	−0.0044 (9)
C11	0.0522 (15)	0.0301 (12)	0.0563 (16)	0.0035 (11)	0.0320 (13)	−0.0021 (11)

### Geometric parameters (Å, °)

**Table:**

Co1—N1	2.1175 (18)	C8—H8A	0.9300
Co1—N1^i^	2.1175 (18)	C1—C2	1.383 (3)
Co1—N2^i^	2.1285 (17)	C1—H1A	0.9300
Co1—N2	2.1285 (17)	C6—C7	1.388 (3)
Co1—O1	2.1420 (15)	C6—C5	1.482 (3)
Co1—O1^i^	2.1420 (15)	C3—C2	1.380 (3)
Co1—S1	2.7122 (9)	C3—C4	1.382 (3)
S1—O2^i^	1.4629 (15)	C3—H3A	0.9300
S1—O2	1.4629 (15)	C9—H9A	0.9300
S1—O1^i^	1.4958 (15)	C4—C5	1.388 (3)
S1—O1	1.4958 (15)	C4—H4A	0.9300
N2—C10	1.339 (3)	C7—H7A	0.9300
N2—C6	1.354 (3)	C2—H2A	0.9300
N1—C1	1.339 (3)	O3—C11	1.398 (3)
N1—C5	1.354 (3)	O3—H3	0.8200
C10—C9	1.384 (3)	C11—C11^i^	1.492 (5)
C10—H10A	0.9300	C11—H11A	0.9700
C8—C7	1.380 (3)	C11—H11B	0.9700
C8—C9	1.384 (3)		
			
N1—Co1—N1^i^	99.06 (9)	N2—C10—C9	122.8 (2)
N1—Co1—N2^i^	95.55 (7)	N2—C10—H10A	118.6
N1^i^—Co1—N2^i^	76.92 (7)	C9—C10—H10A	118.6
N1—Co1—N2	76.92 (7)	C7—C8—C9	119.5 (2)
N1^i^—Co1—N2	95.55 (7)	C7—C8—H8A	120.3
N2^i^—Co1—N2	168.54 (9)	C9—C8—H8A	120.3
N1—Co1—O1	158.26 (6)	N1—C1—C2	123.0 (2)
N1^i^—Co1—O1	98.95 (6)	N1—C1—H1A	118.5
N2^i^—Co1—O1	100.31 (6)	C2—C1—H1A	118.5
N2—Co1—O1	89.31 (6)	N2—C6—C7	121.38 (19)
N1—Co1—O1^i^	98.95 (6)	N2—C6—C5	115.13 (17)
N1^i^—Co1—O1^i^	158.26 (6)	C7—C6—C5	123.48 (18)
N2^i^—Co1—O1^i^	89.31 (6)	C2—C3—C4	118.8 (2)
N2—Co1—O1^i^	100.31 (6)	C2—C3—H3A	120.6
O1—Co1—O1^i^	66.68 (8)	C4—C3—H3A	120.6
N1—Co1—S1	130.47 (5)	C10—C9—C8	118.3 (2)
N1^i^—Co1—S1	130.47 (5)	C10—C9—H9A	120.8
N2^i^—Co1—S1	95.73 (5)	C8—C9—H9A	120.8
N2—Co1—S1	95.73 (5)	C3—C4—C5	119.7 (2)
O1—Co1—S1	33.34 (4)	C3—C4—H4A	120.1
O1^i^—Co1—S1	33.34 (4)	C5—C4—H4A	120.1
O2^i^—S1—O2	111.03 (13)	N1—C5—C4	121.42 (18)
O2^i^—S1—O1^i^	110.97 (9)	N1—C5—C6	115.52 (17)
O2—S1—O1^i^	109.91 (8)	C4—C5—C6	123.05 (18)
O2^i^—S1—O1	109.91 (8)	C8—C7—C6	119.3 (2)
O2—S1—O1	110.97 (9)	C8—C7—H7A	120.4
O1^i^—S1—O1	103.82 (12)	C6—C7—H7A	120.4
O2^i^—S1—Co1	124.48 (6)	C3—C2—C1	118.8 (2)
O2—S1—Co1	124.48 (6)	C3—C2—H2A	120.6
O1^i^—S1—Co1	51.91 (6)	C1—C2—H2A	120.6
O1—S1—Co1	51.91 (6)	C11—O3—H3	109.5
S1—O1—Co1	94.75 (8)	O3—C11—C11^i^	113.9 (2)
C10—N2—C6	118.71 (18)	O3—C11—H11A	108.8
C10—N2—Co1	125.17 (14)	C11^i^—C11—H11A	108.8
C6—N2—Co1	116.09 (13)	O3—C11—H11B	108.8
C1—N1—C5	118.22 (17)	C11^i^—C11—H11B	108.8
C1—N1—Co1	125.50 (14)	H11A—C11—H11B	107.7
C5—N1—Co1	116.26 (13)		
			
N1—Co1—S1—O2^i^	113.14 (10)	O1^i^—Co1—N2—C6	−99.02 (14)
N1^i^—Co1—S1—O2^i^	−66.86 (10)	S1—Co1—N2—C6	−132.39 (13)
N2^i^—Co1—S1—O2^i^	10.83 (9)	N1^i^—Co1—N1—C1	88.69 (17)
N2—Co1—S1—O2^i^	−169.17 (9)	N2^i^—Co1—N1—C1	11.08 (18)
O1—Co1—S1—O2^i^	−89.23 (11)	N2—Co1—N1—C1	−177.68 (18)
O1^i^—Co1—S1—O2^i^	90.77 (11)	O1—Co1—N1—C1	−125.71 (19)
N1—Co1—S1—O2	−66.86 (10)	O1^i^—Co1—N1—C1	−79.09 (18)
N1^i^—Co1—S1—O2	113.14 (10)	S1—Co1—N1—C1	−91.31 (17)
N2^i^—Co1—S1—O2	−169.17 (9)	N1^i^—Co1—N1—C5	−93.12 (14)
N2—Co1—S1—O2	10.83 (9)	N2^i^—Co1—N1—C5	−170.72 (14)
O1—Co1—S1—O2	90.77 (11)	N2—Co1—N1—C5	0.52 (13)
O1^i^—Co1—S1—O2	−89.23 (11)	O1—Co1—N1—C5	52.5 (2)
N1—Co1—S1—O1^i^	22.38 (9)	O1^i^—Co1—N1—C5	99.11 (14)
N1^i^—Co1—S1—O1^i^	−157.62 (9)	S1—Co1—N1—C5	86.88 (14)
N2^i^—Co1—S1—O1^i^	−79.94 (9)	C6—N2—C10—C9	−1.1 (3)
N2—Co1—S1—O1^i^	100.06 (9)	Co1—N2—C10—C9	176.85 (16)
O1—Co1—S1—O1^i^	180.0	C5—N1—C1—C2	0.3 (3)
N1—Co1—S1—O1	−157.62 (9)	Co1—N1—C1—C2	178.49 (16)
N1^i^—Co1—S1—O1	22.38 (9)	C10—N2—C6—C7	0.9 (3)
N2^i^—Co1—S1—O1	100.06 (9)	Co1—N2—C6—C7	−177.24 (16)
N2—Co1—S1—O1	−79.94 (9)	C10—N2—C6—C5	−178.55 (18)
O1^i^—Co1—S1—O1	180.0	Co1—N2—C6—C5	3.3 (2)
O2^i^—S1—O1—Co1	118.76 (8)	N2—C10—C9—C8	0.8 (3)
O2—S1—O1—Co1	−118.03 (8)	C7—C8—C9—C10	−0.3 (3)
O1^i^—S1—O1—Co1	0.0	C2—C3—C4—C5	0.1 (3)
N1—Co1—O1—S1	51.43 (19)	C1—N1—C5—C4	0.6 (3)
N1^i^—Co1—O1—S1	−162.95 (7)	Co1—N1—C5—C4	−177.70 (14)
N2^i^—Co1—O1—S1	−84.73 (8)	C1—N1—C5—C6	179.33 (18)
N2—Co1—O1—S1	101.54 (8)	Co1—N1—C5—C6	1.0 (2)
O1^i^—Co1—O1—S1	0.0	C3—C4—C5—N1	−0.9 (3)
N1—Co1—N2—C10	179.84 (18)	C3—C4—C5—C6	−179.46 (19)
N1^i^—Co1—N2—C10	−82.12 (18)	N2—C6—C5—N1	−2.8 (3)
N2^i^—Co1—N2—C10	−130.43 (17)	C7—C6—C5—N1	177.70 (18)
O1—Co1—N2—C10	16.80 (17)	N2—C6—C5—C4	175.85 (17)
O1^i^—Co1—N2—C10	82.94 (18)	C7—C6—C5—C4	−3.6 (3)
S1—Co1—N2—C10	49.57 (17)	C9—C8—C7—C6	0.1 (3)
N1—Co1—N2—C6	−2.12 (13)	N2—C6—C7—C8	−0.4 (3)
N1^i^—Co1—N2—C6	95.92 (14)	C5—C6—C7—C8	179.00 (19)
N2^i^—Co1—N2—C6	47.61 (13)	C4—C3—C2—C1	0.8 (3)
O1—Co1—N2—C6	−165.15 (14)	N1—C1—C2—C3	−1.0 (3)

Symmetry codes: (i) −*x*, *y*, −*z*+1/2.

### Hydrogen-bond geometry (Å, °)

**Table:**

*D*—H···*A*	*D*—H	H···*A*	*D*···*A*	*D*—H···*A*
O3—H3···O2	0.82	1.97	2.758 (2)	160.
